# Overexpression of Extracellular Superoxide Dismutase 3 Inhibits Cancer Cell Growth and Migration in Colorectal Cancer

**DOI:** 10.5152/tjg.2024.23232

**Published:** 2024-06-01

**Authors:** Donghua Wang, Manyu Chen, Zhenggui Tao, Jinghu Du, Kui Tian, Zhen Chen, Bo Yu, Yu Chen, Long Lv

**Affiliations:** 1 Department of Coloproctological Surgery, Xiangyang Central Hospital, Affiliated Hospital of Hubei University of Arts and Science, Xiangyang, China; 2 Department of Emergency, The Central Hospital of Wuhan, Tongji Medical College, Huazhong University of Science and Technology, Wuhan, China

**Keywords:** Colorectal cancer, extracellular superoxide dismutase, metastasis, epithelial–mesenchymal transition, prognosis

## Abstract

**Background/Aims:**

Incidence of colorectal cancer is rapidly increasing worldwide. Extracellular superoxide dismutase (EcSOD; SOD3) is an antioxidant enzyme. However, SOD3 roles in colorectal cancer progression remain uncertain.

**Materials and Methods:**

Superoxide dismutase 3 expression was evaluated, and we analyzed clinical relevance of SOD3 expression in colorectal cancer. Subsequently, SOD3 roles in colorectal cancer progression were detected by gain of function experiments. Changes in subcutaneous tumor and liver nodule size after SOD3 overexpression were examined in nude mice. The expression of proliferation marker Ki67 was assessed by immunohistochemical staining.

**Results:**

Supperoxide dismutase 3 was downregulated in colorectal cancer (*P* < .01). Downregulation of SOD3 was correlated with unfavorable outcomes (*P* < .05). Superoxide dismutase 3 upregulation limited the proliferative (*P* < .05), migrative (*P* < .01) and invasive actions of colorectal cancer cells (*P* < .01) by suppressing epithelial–mesenchymal transition. Moreover, SOD3 overexpression reduced Ki67 expression (*P* < .01) and blocked tumor growth (*P* < .01) and liver metastasis (*P* < .001) in mouse tumor model.

**Conclusion:**

Superoxide dismutase 3 upregulation attenuates tumor growth and liver metastasis in colorectal cancer, suggesting that SOD3 has potential diagnostic and prognostic values regarding colorectal cancer treatment.

**Main Points**

- Superoxide dismutase 3 (SOD3) levels are downregulated and their downregulation exhibits unfavorable outcomes in colorectal cancer.
- Overexpression of SOD3 prevents colorectal cancer cell proliferation.
- Overexpression of SOD3 prevents the malignant phenotypes of colorectal cancer cells through inhibition of epithelial–mesenchymal transition.
- Elevation of SOD3 limits tumor growth and reduces Ki67 expressionin vivo.
- Overexpression of SOD3 suppresses liver metastasis in animal models.

## Introduction

Colorectal cancer (CRC) is increasing in the incidence. In 2020, approximately 1.93 million people were newly diagnosed with CRC, and there were approximately 930 000 deaths worldwide.^1^ Colorectal cancer development is attributed to age, genetic and environmental factors.^2-4^ Currently, CRC is commonly treated with colectomy, chemotherapy, and immunotherapy. However, approximately 35%-55% of patients with CRC. still develop liver metastasis, which seriously affects prognosis in patients with CRC.^5,6^ Colorectal cancer is easily misdiagnosed due to its asymptomatic nature in the early stages, which leads to diagnosis at advanced disease stages, increasing the risk of tumor metastasis.^7^ The predominant contributor to CRC-related death for 90% of patients is tumor metastasis, which is strongly with epithelial–mesenchymal transition (EMT).^8^ Thus, the investigation of novel targets that can modulate EMT is beneficial for improving CRC treatment and prognosis.

Heparanase can cleave heparan sulfate side chains to modify the extracellular matrix integrity, enhancing tumor cell invasion. Cleavage of heparan sulfate by heparanase also induces the secretion of bioactive cytokines, promoting tumor growth.^9^ In addition to heparanase, reactive oxygen species (ROS) are found to exert effects on heparan sulfate degradation. Superoxide dismutase (SOD), a cellular antioxidant, can convert superoxide radicals into hydrogen peroxide, providing the primary defense against ROS.^10^ Scavenging ROS with SOD contributes to improving heparan integrity and blocking heparanase-mediated tumor development.^11^ Extracellular SOD (EcSOD; SOD3) has been found to bind to heparan sulfate in the extracellular environment and can protect heparan sulfate from fragmentation induced by oxidative species.^12^ Emerging evidence implicates SOD3 downregulation in many cancers, including pancreatic ductal adenocarcinoma,^13^ lung cancer^14^ and prostate cancer.^15^ Simultaneously, overexpression of SOD3 can inhibit tumor growth and metastasis.^16^ As reported, SOD3 expression is significantly decreased in CRC tumor samples.^17^ Nevertheless, the potential function of SOD3 in CRC progression is still unknown. Therefore, we investigated the association between SOD3 expression and CRC progression. We hypothesized that SOD3 might inhibit CRC development, and it might be a potential target for CRC treatment.

## Materials and Methods

### Patients

This study included 80 CRC patients who had undergone radical resection at Xiangyang Central Hospital, Affiliated Hospital of Hubei University of Arts and Science from January 2018 to July 2021. Patients with newly diagnosed CRC who didn’t receive chemotherapy or radiotherapy were included. However, patients with other clinical dysfunction, recurrent CRC, or those who died of unrelated reasons were excluded from the study. We performed a 5-year follow-up study on the survival conditions of these patients. All participants signed informed consent. The protocol of this study was approved by the Ethics Committee of Xiang Yang Central Hospital (approval number: KY-20220114-003, date: January 14, 2022). The tumor and adjacent non-tumor tissues (within 2 cm around tumors) were collected.

### Cell Culture

Human CRC cell lines (SW620, SW480, DLD-1, and HCT116) and normal intestinal epithelial cell line (NCM460) acquired from Sunncell (Wuhan, China) were cultured in RPMI-1640 medium containing 10% fetal bovine serum (FBS) and 1% penicillin–streptomycin (Sigma-Aldrich, Shanghai, China) at 37°C with 5% CO_2_ under humidified conditions.

### Gene Transfer into Colorectal Cancer Cells

The adenovirus infection method was used to overexpress SOD3 as previously described.^18^ DLD-1, HCT116, SW480, and SW620 cells were seeded on 6-well plates (1.5 × 10^5^ cells/well), incubated for 2 hours, and infected with the Ad-SOD3-EGFP (overexpression vector) or Ad-EGFP (empty vector) for 48 hours. The experimental group was as follows: Ad-EGFP group (vector) and Ad-SOD3-EGFP group (SOD3).

### Real Time-Quantitative Polymerase Chain Reaction

Total RNA was isolated via a TRIzol reagent (Sigma-Aldrich), and its concentration was examined using a NanoDrop 1000 spectrophotometer (ThermoFisher Scientific, Shanghai, China). Then, RNA (1 mg) was reverse-transcribed into cDNA using a PrimeScript RT reagent kit (Jinpan Biotech, Shanghai, China). Real time quantitative polymerase chain reaction (RT-qPCR) was performed on an ABI 7500 instrument. Glyceraldehyde-3-phosphate dehydrogenase (GAPDH) served as an internal reference for mRNA detection. Quantification was conducted using the 2^−△△^^Ct^ method.^19^ The primer sequences were listed in Table 1. The correlation between SOD3 expression and clinicopathological characteristics of CRC was listed in Table 2.

### Western Blotting

Total protein was extracted by RIPA lysis buffer (MedChemExpress, Shanghai, China) containing protease inhibitor cocktails and phosphatase inhibitor cocktails and centrifuged at 12 000 *g* for 10 minutes at 4°C. Then, quantification of protein concentration was performed via a BCA Protein Assay Kit (Beyotime). Next, protein samples (20 μg/group) were separated by SDS-PAGE and transferred onto PVDF membranes. After blocking using 5% skimmed milk, the membranes were incubated overnight with anti-N-cadherin (ab76011, 1:5000), anti-SOD3 (ab80946, 1:1000), anti-GAPDH (ab8245, 1:2000), anti-E-cadherin (ab40772, 1:2000), and anti-vimentin (ab92547, 1:3000) primary antibodies at 4°C and then incubated with secondary antibodies for 2 hours. Antibodies were acquired from Abcam (Shanghai, China). The immunoblots were visualized using an enhanced chemiluminescence reagent (Yeasen) and analyzed by ImageJ software.

### Wound Healing Assays

Cell migration was assessed as previously described.^20^ Cells were incubated in 6-well plates (2 × 10^5^ cells/well) for 12 hours. When growing to 100% confluence, the cell monolayer was scratched via a 10 μL pipette tip. Then, the cells were incubated in serum-free medium at 37°C, and imaged at 0 and 24 hours by an inverted microscope (Olympus, Tokyo, Japan).

### Cell counting kit-8 (CCK-8)

The DLD-1, HCT116, SW480, and SW620 cells seeded in 96-well plates (2 × 10^4^ cells/well) were incubated at 37°C with 5% CO_2_ for 24, 48, 72, and 96 hours prior to the addition of 10 μL CCK-8 solution (Yeasen) to each well. After 2 hours, a spectrophotometer (Molecular Devices, Shanghai, China) was used to read the absorbance at 450 nm.

### Cell Proliferation Assays

Cell proliferation was assessed as previously described.^21^ Briefly, 2 × 10^3^ cells were added to RPMI-1640 medium containing 0.35% low-melting agarose and 20% FBS, and then the mixture was layered on top of the base layer of 0.7% low-melting agarose. The cells were then inoculated into 6-well plates. After 2 weeks, colonies were subjected to 0.4% crystal violet staining and imaged via a digital camera (Nikon, Tokyo, Japan).

### Transwell Assay

Cell motion was assessed through Transwell chambers.^20^ The cells (5 × 10^4^) suspended in 200 μL serum-free medium were incubated in the upper chambers for 24 hours for migration assays. The upper chambers were precoated with Matrigel (Sigma-Aldrich), and 600 μL medium containing 10% FBS was added to the lower chamber and incubated for 48 hours for invasion assays. After removal of non-migrating or non-invasive cells, the migrated or invaded cells were fixed with 4% paraformaldehyde for 20 minutes, stained with crystal violet for 15 minutes, and counted by a light microscope.

### Tumor Xenograft Model

Tumor xenograft model was established using a previously described method.^22^ BALB/c nude mice (male, 4 weeks; Cyagen, Suzhou, China) were raised under pathogen-free conditions with free food and water and randomly divided as follows: vector (DLD-1) group, SOD3 (DLD-1) group, vector (HCT116) group, and SOD3 (HCT116) group. DLD-1 and HCT116 cells carrying SOD3 overexpression or empty vector were suspended in PBS (5 × 10^6^ cells in 0.1 mL PBS) and subcutaneously injected into the right dorsal flanks of mice. Each group had 4 mice. On day 21 post operation, mice were sacrificed by cervical dislocation, and tumor tissues were harvested and weighed. All animal experiments were approved by the Laboratory Animal Ethics Committee of Hubei University of Arts and Science (approval number: HDRM20230201-1, date: February 1, 2023).

### Immunohistochemical Staining

Immunohistochemical staining was performed using a previously documented method.^23^ Briefly, paraffin-embedded tissues were sectioned into 4 μm slices, dewaxed and rehydrated. After incubation in 3% H_2_O_2_ at room temperature for 15 minutes and boiling in 0.01 M citrate buffer (pH 6.0), the slices were incubated with the primary antibody against the proliferation marker Ki67 (ab15580, 1:100; Abcam) overnight at 4°C and then incubated with biotin-labeled secondary antibody at room temperature for 1 hour. Positive signal of target protein was amplified by streptavidin and biotin-labeled horseradish peroxidase in SABC-HRP kit (Beyotime) and observed using an optical microscope (Olympus).

### Liver Metastasis Assay

BALB/c mice (5 mice in each group) were subjected to intrasplenic injection of DLD-1 or HCT116 cells (2 × 10^6^) that suspended in PBS and sacrificed after 5 weeks.^24^ Their liver specimens were harvested, fixed in 10% paraformaldehyde, paraffin-embedded, and cut into 5 μm slices. After dewaxing and rehydrating, the liver slices were stained with hematoxylin and eosin (Sigma-Aldrich) and imaged via a light microscope (Olympus).

### Statistical Analysis

All data was averaged from 3 independent experiments. Two observers who were blinded to the experimental design participated in statistical analysis. All results were analyzed by GraphPad Prism and reported as the mean ± SD. One-way analysis of variance followed by Tukey’s post hoc analysis and Student’s *t*-test were used for statistical analysis. *P* < .05 was considered statistically significant.

## Results

### Superoxide Dismutase Is Downregulated in Colorectal Tissues and Cells and Its Downregulation Predicts a Poor Prognosis in Patients with Colorectal Cancer

**Figure 1.**
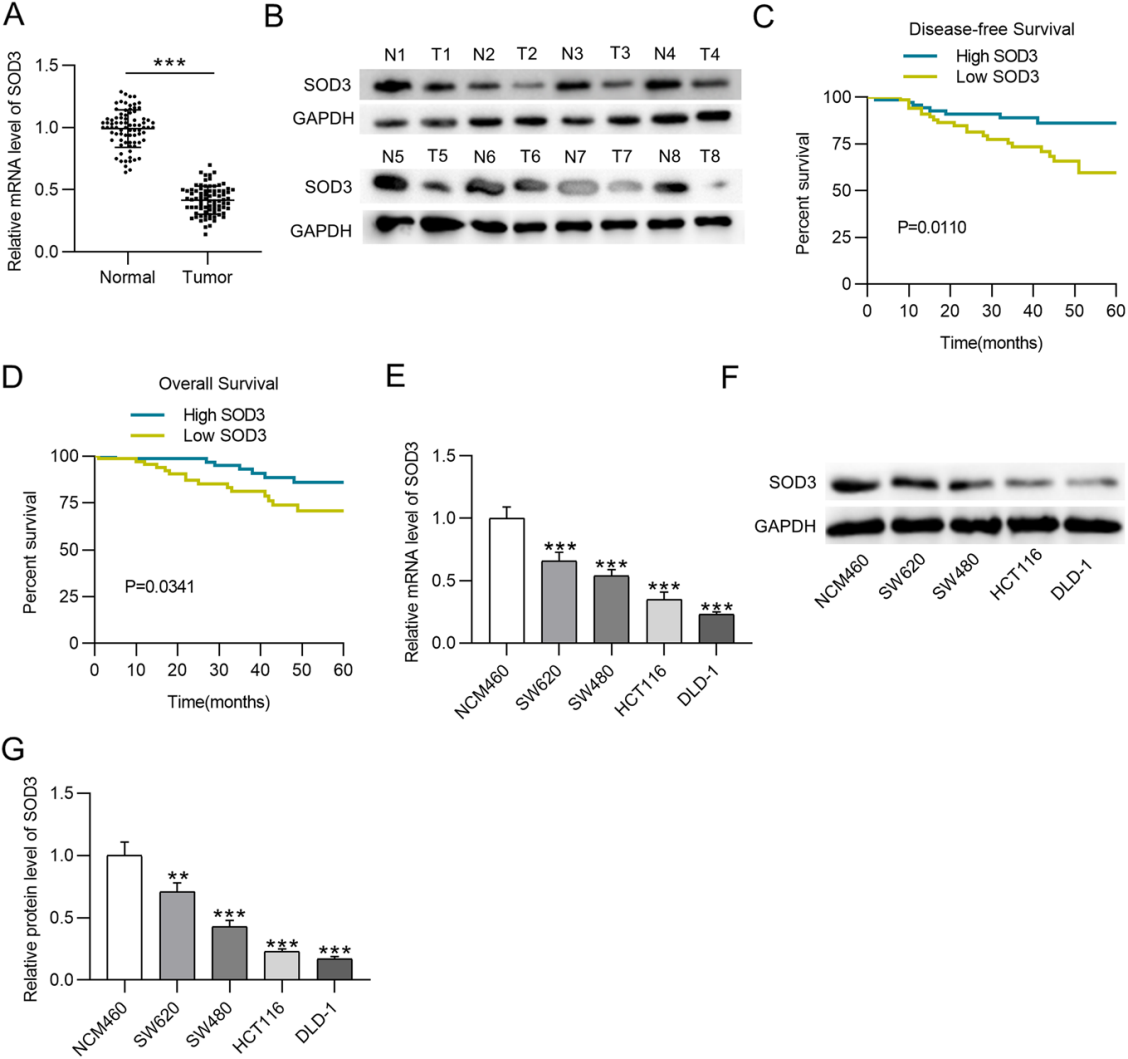
SOD3 is downregulated in CRC tissues and cells and its downregulation predicts a poor prognosis. (A) RT-qPCR to measure the mRNA level of SOD3 in 80 paired CRC and adjacent normal tissues. (B) Western blotting to measure the protein level of SOD3 in 8 randomly selected paired tissues. (C and D) Overall survival and disease-free survival analysis according to the expression of SOD3. (E-G) RT-qPCR and western blotting to evaluate the mRNA and protein levels of SOD3 in the NCM460 cell line and CRC cell lines (SW620, SW480, DLD-1, and HCT116). Data are shown as mean ± SD. **P < .01, ***P < .001. SOD3, superoxide dismutase 3; RT-qPCR, real-time quantitative polymerase chain reaction.

Colorectal cancer tumor tissues exhibited a higher SOD3 mRNA level than adjacent normal tissues (Figure 1A). Then, the SOD3 protein level in 8 pairs of randomly chosen CRC tumor and adjacent normal tissues was measured through western blotting. The SOD3 protein level was statistically downregulated in CRC tumor samples (Figure 1B). In CRC patients, SOD3-low expression was significantly related to T and N stages and tumor differentiation (Table 2). In parallel, SOD3-low expression was correlated with an unfavorable prognostic outcomes (Figure 1C and 1D). Next, the expression of SOD3 in SW620, SW480, DLD-1, HCT116, and NCM460 was evaluated via RT-qPCR and western blotting. We found statistically downregulated SOD3 mRNA and protein levels in CRC cells (Figure 1E-G). Collectively, SOD3 is downregulated in CRC, and its downregulation predicts a poor prognostic outcomes.

### Superoxide Dismutase 3 Overexpression Inhibits Colorectal Cancer Cell Viability and Proliferation

**Figure 2.**
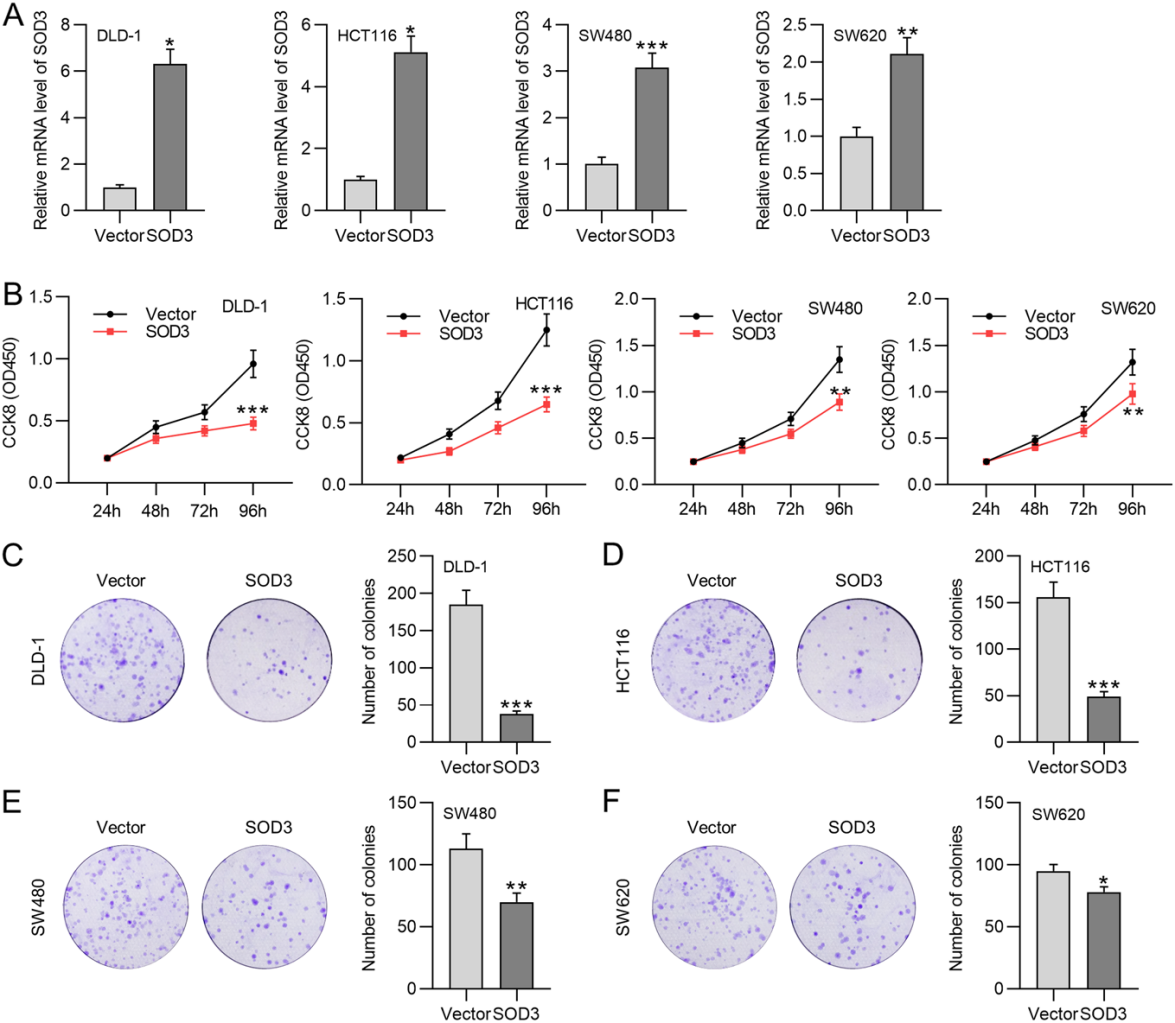
Supeoxide dismutase 3 elevation blocks CRC cell proliferation. (A) RT-qPCR was performed to detect the overexpression efficiency of the SOD3 overexpression vector in DLD-1, HCT116, SW480, and SW620 cells. (B) CCK-8 assays were conducted to assess cell viability. (C-F) Colony formation assays were used to evaluate cell proliferation. Data are shown as mean ± SD. *P < .05, **P < .01, ***P < .001.

Due to the downregulation of SOD3 in CRC cells, we effectively overexpressed SOD3 expression in the DLD-1, HCT116, SW480, and SW620 cell lines (Figure 2A). Superoxide dismutase 3 upregulation limited CRC cell proliferation (Figure 2B-F). Subsequently, we used DLD-1 and HCT116 cells due to the more effective viability and proliferation. inhibitory effects of SOD3 upregulation on DLD-1 and HCT116 cells than the other 2 cell lines.

### Superoxide Dismutase 3 Overexpression Limits Malignant Phenotypes of Colorectal Cancer Cells by Regulating Epithelial–Mesenchymal Transition

**Figure 3.**
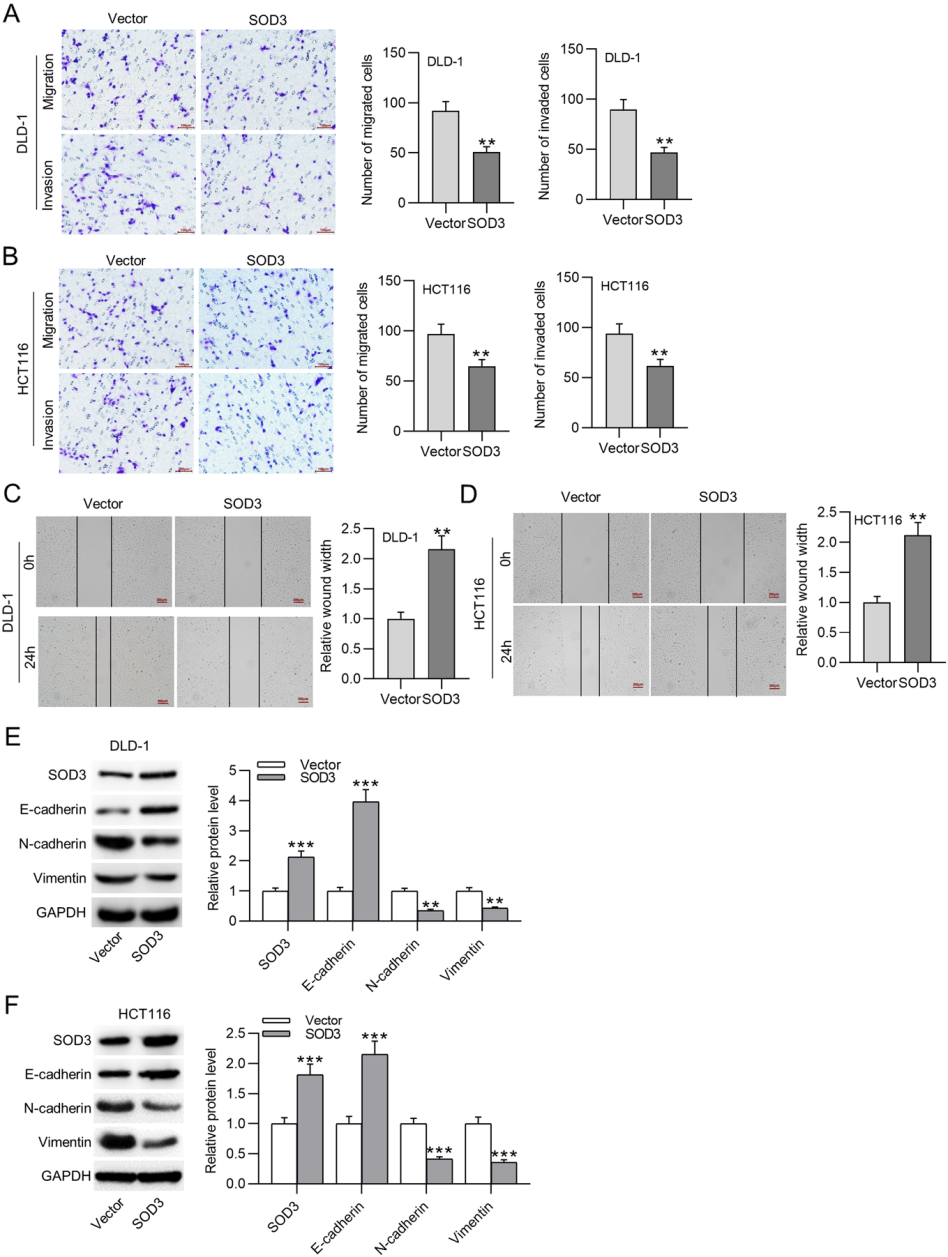
Superoxide dismutase 3 overexpression limits CRC cell malignancies by regulating epithelial–mesenchymal transition. (A-D) Transwell assays and wound healing assays to assess cell migration and invasion. (E and F) Western blotting of SOD3, E-cadherin, N-cadherin, and Vimentin protein levels. Data are shown as mean ± SD. **P < .01, ***P < .001. CRC, colorectal cancer; SOD3, superoxide dismutase 3.

To verify if SOD3 overexpression attenuated the metastatic capability of CRC cells, Transwell assays were conducted. Superoxide dismutase 3 upregulation markedly inhibited cancer cell migration and invasion (Figure 3A and 3B). Then, wound healing assays confirmed the migratory suppressive effect of SOD3 upregulation (Figure 3C and 3D). Moreover, as western blotting revealed, SOD3 overexpression notably increased E-cadherin protein level while downregulating N-cadherin and Vimentin protein levels in CRC cells (Figure 3E and 3F). The above findings suggest that SOD3 upregulation suppresses CRC cell metastasis by inhibiting EMT.

### Superoxide Dismutase 3 Overexpression Inhibits Colorectal Cancer Tumor Growth

**Figure 4.**
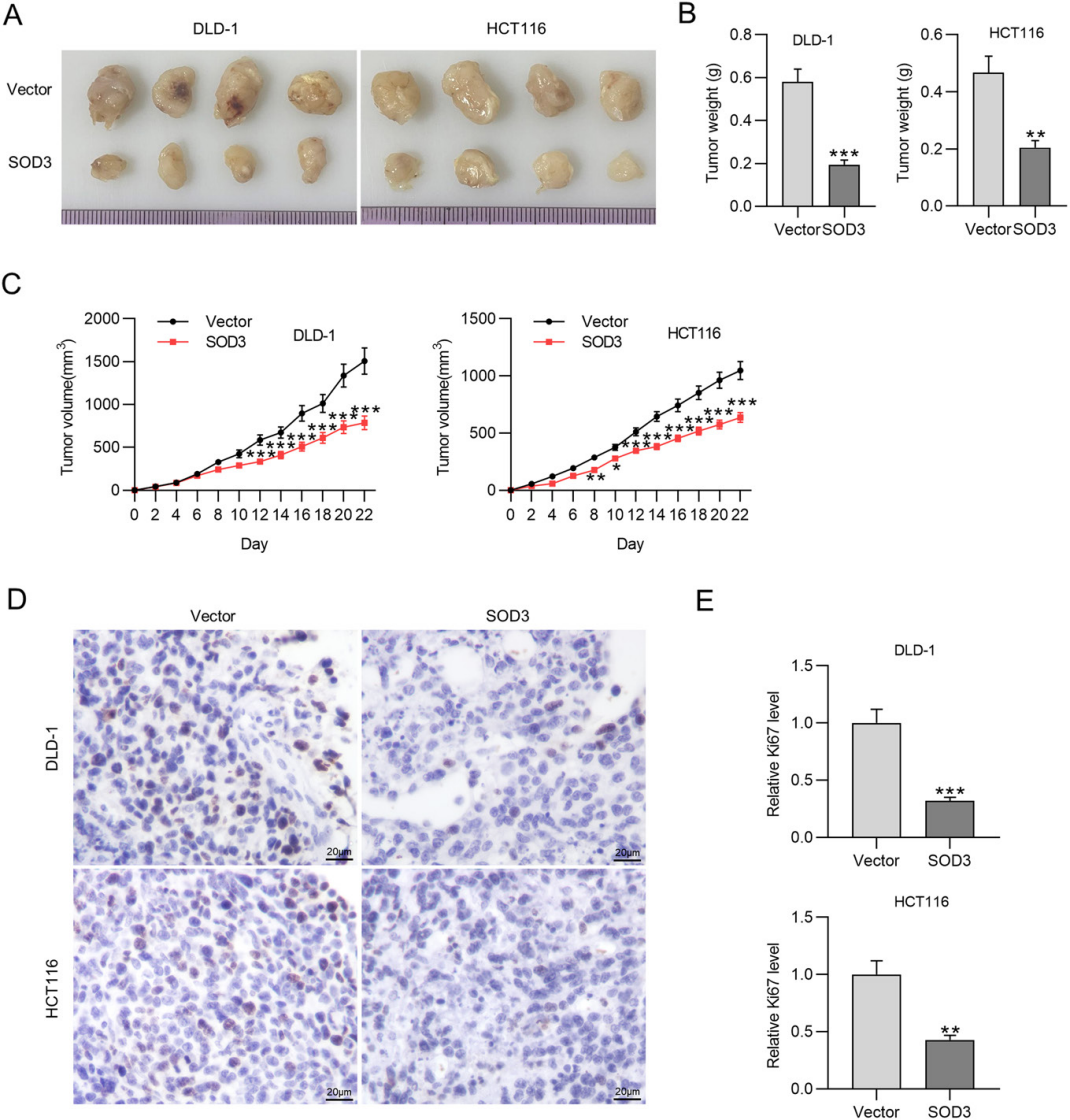
SOD3 overexpression inhibits CRC tumor growth. (A) Macroscopic images of resected tumors. (B) Tumor weight. (C) Tumor volume. (D) Immunohistochemical analysis of Ki67 expression. (E) Quantitative analysis of Ki67 expression. Data are shown as mean ± SD. *P < .05, **P < .01, ***P < .001. SOD3, superoxide dismutase 3.

Next, we established a classical xenograft mouse model to further assess the potential antitumorigenic effect of SOD3 overexpression. Tumor tissues in different groups were presented in Figure 4A. Tumor weight and size were remarkably decreased in SOD3 overexpressing groups (Figure 4B and 4C). Ki67 serves as a marker of cell proliferation, and its expression was measured by immunohistochemistry staining. Figure 4D presents the representative images of Ki67 staining. The Ki67 quantification suggested that SOD3 elevation inhibited tumor cell proliferation (Figure 4E). These results indicate that SOD3 overexpression effectively inhibits tumor growth and CRC proliferation in mice.

### Superoxide Dismutase 3 Overexpression Suppresses Colorectal Liver Metastasis

**Figure 5.**
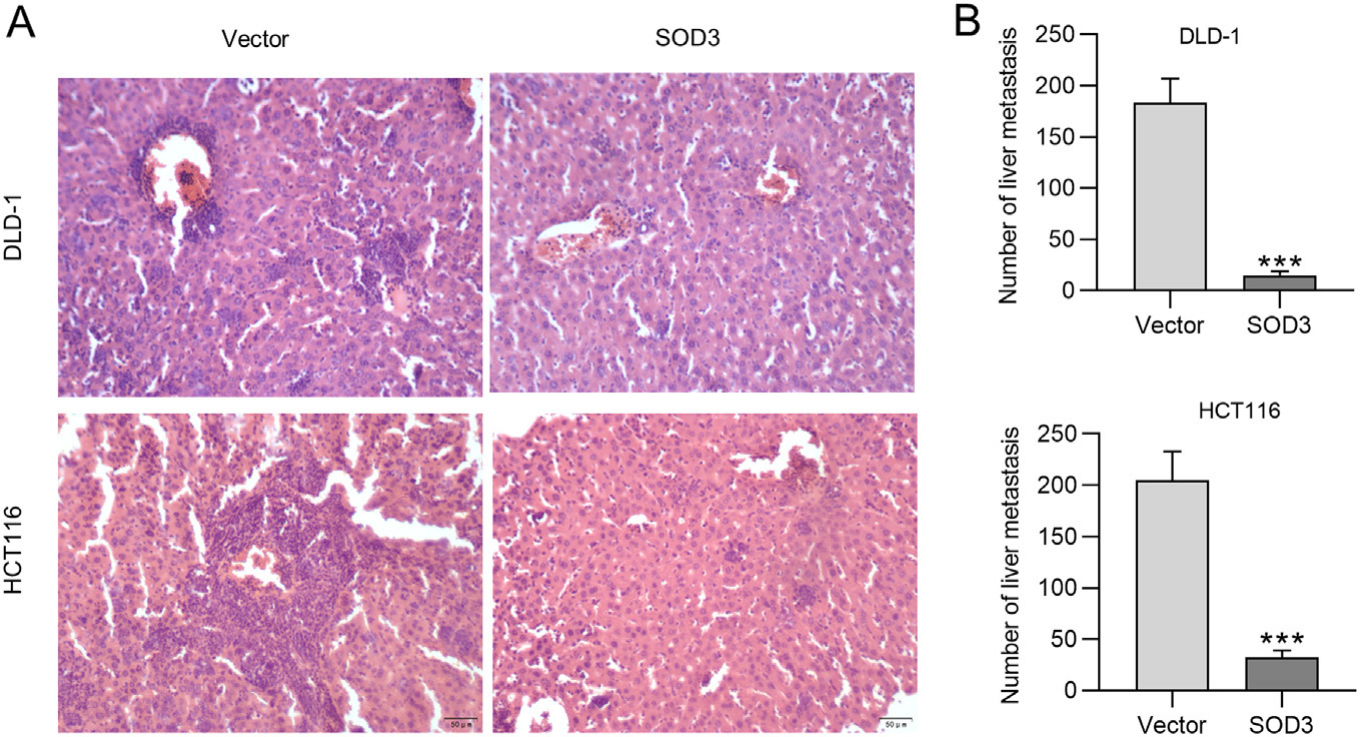
SOD3 overexpression suppresses colorectal cancer liver metastasis. (A) Microscopic images of resected livers stained by H&E. (B) Quantification of the number of liver metastatic nodules. Data are shown as mean ± SD. ***P < .001. SOD3, superoxide dismutase 3.

Male BALB/c nude mice were subjected to intrasplenic injections of cancer cells and sacrificed after 5 weeks. Hematoxylin and eosin-stained livers of SOD3-overexpressed mice displayed reduced metastatic nodules (Figure 5A and 5B). These results support that thein vitrometastasis suppressing effect of SOD3 overexpression is also presentin vivo.

## Discussion

Colorectal cancer is a malignant neoplasm. Despite great advancements in early detection and management, the prognosis in CRC patients remains unfavorable. Thus, exploration of novel effective targets with diagnostic value for CRC is urgent. Accumulating evidence has suggested that ROS is highly expressed in CRC cells.^25,26^ It has been demonstrated that ROS elevation can lead to genetic instability and cancer progression.^27^ SODs function as a defense mechanism against ROS. There are 3 isoforms of SOD, including CuZn-SOD (SOD1), MnSOD (SOD2), and EcSOD (SOD3).^10^ Both SOD1 and SOD2 have been extensively studied in CRC, but information regarding SOD3 role in CRC progression is still limited. Herein, we found SOD3 downregulation in tumor tissues and its downregulation predicted poor outcomes in CRC. Concomitantly, CRC cells also exhibited lower SOD3 expression than normal cells, and SOD3 upregulation suppressed CRC metastasis and tumor growth.

A previous study regarding the association between SOD3 polymorphism and a high susceptibility to CRC suggests that there is no statistical association between SOD3 polymorphism and susceptibility to CRC.^28^ However, the association between SOD3 and CRC progression has been previously reported. Superoxide dismutase 3 levels are downregulated in CRC tissues,^17,29^ and SOD3 positivity is associated with lower recurrent rates.^30^ However, the specific functions of SOD3 in CRC development remain uncertain. This was the first research to investigate the specific functions of SOD3 in CRC.

Downregulated SOD3 levels have been found in breast, lung, and pancreas carcinoma.^13,31,32^ Loss of SOD3 expression can also contribute to tumor recurrence and poor patient outcome.^33,34^ Herein, SOD3 was downregulated in CRC, and its low expression was positively correlated with an unfavorable prognosis. Epithelial–mesenchymal transition is related to the invasion and metastasis of CRC.^35-37^ Superoxide dismutase 3 deficiency can promote hepatic stellate cell activation and EMT to induce liver fibrogenesis.^38^ Additionally, SOD3 upregulation prevents the expression of mesenchymal proteins.^39^ Herein, we found that elevated SOD3 levels elevated E-cadherin levels while decreasing N-cadherin and Vimentin levels in CRC cells, indicating that SOD3 upregulation prevents EMT in CRC. Moreover, SOD3 overexpression can inhibit cell growth and metastasis in prostate, pancreatic, thyroid, and lung cancers,^15,32,40,41^ and also inhibits tumor metastasis in a metastasis mouse model.^42^ Herein, SOD3 overexpression limited the proliferative, migrative, and invasive capabilities of CRC cells and also prevented tumor growth and liver metastasis.

There are limitations to this study. First, loss-of-function experiments are required to confirm SOD3’s role in CRC. Second, the underlying mechanisms by which SOD3 affects CRC should be further investigated. Third, more biological functions of SOD3 in CRC need to be detected. Despite these certain limitations, our findings represent a valuable contribution to the literature and may help develop novel treatments for CRC.

In conclusion, this study demonstrated that SOD3 upregulation inhibited CRC cell growth and metastasis and also limited tumor growth and liver metastasisin vivo.

## Figures and Tables

**Table 1.** Sequences of Primers Used for Reverse Transcription Quantitative Polymerase Chain Reaction

Gene (Human)	Sequence (5’⟶3’)
SOD3 forward	CATGCAATCTGCAGGGTACAA
SOD3 reverse	AGAACCAAGCCGGTGATCTG
GAPDH forward	ATATTGTTGCCATCAATGACC
GAPDH reverse	ATATTGTTGCCATCAATGACC

GAPDH, glyceraldehyde 3-phosphate dehydrogenase; SOD3, superoxide dismutase 3.

**Table 2.** Correlation Between Superoxide Dismutase 3 Expression and Clinicopathological Characteristics of Colorectal Cancer

Characteristics		Cases	SOD3 Low (39)	SOD3 High (41)	*P*
Gender	Male	51	24	27	.869
	Female	29	15	14	
Age	<60	37	17	20	.642
	≥60	43	22	21	
Tumor location	Colon	53	26	27	.939
	Rectum	27	13	14	
T stage	T1-2	48	16	32	.001
	T3-4	32	23	9	
N stage	N0	43	15	28	.007
	N1-2	37	24	13	
M stage	M0	45	20	25	.382
	M1	35	19	16	
Differentiation	Low	16	5	11	.038
	Medium	48	22	26	
	High	16	12	4	
Size	<4.5 cm	41	20	21	.996
	≥4.5 cm	39	19	20	

## References

[1] Cancer international agency for research. Latest global cancer data: cancer burden rises to 19.3 million new cases and 10.0 million cancer deaths in 2020; 2020. https://www.iarc.fr/fr/news-events/latest-global-cancer-data-cancer-burden-rises-to-19-3-million-new-cases-and-10-0-million-cancer-deaths-in-2020/

[2] Thanikachalam K Khan G . Colorectal cancer and nutrition. Nutrients. 2019;11(1) (10.3390/nu11010164) PMC6357054 30646512

[3] Osman S Kahraman Çetin N Erdoğdu İH Meteoğlu İ . Mutation profile via next-generation sequencing in patients with colorectal adenocarcinoma and its clinicopathological correlation. Turk J Gastroenterol. 2023;34(11):1124 1133. (10.5152/tjg.2023.22682) 37737217 PMC10724784

[4] Oukkal M Bouzid K Bounedjar A , et al. Middle East and North Africa registry to characterize rate of RAS testing status in newly diagnosed patients with metastatic colorectal cancer. Turk J Gastroenterol. 2023;34(2):118 127. (10.5152/tjg.2022.22106) 36445057 PMC10081134

[5] Johdi NA Sukor NF . Colorectal cancer immunotherapy: options and strategies. Front Immunol. 2020;11:1624. (10.3389/fimmu.2020.01624) 33042104 PMC7530194

[6] Akgül Ö Çetinkaya E Ersöz Ş Tez M . Role of surgery in colorectal cancer liver metastases. World J Gastroenterol. 2014;20(20):6113 6122. (10.3748/wjg.v20.i20.6113) 24876733 PMC4033450

[7] Li C Sun YD Yu GY , et al. Integrated omics of metastatic colorectal cancer. Cancer Cell. 2020;38(5):734 747.e9. (10.1016/j.ccell.2020.08.002) 32888432

[8] Saitoh M . Involvement of partial EMT in cancer progression. J Biochem. 2018;164(4):257 264. (10.1093/jb/mvy047) 29726955

[9] Weissmann M Arvatz G Horowitz N , et al. Heparanase-neutralizing antibodies attenuate lymphoma tumor growth and metastasis. Proc Natl Acad Sci U S A. 2016;113(3):704 709. (10.1073/pnas.1519453113) 26729870 PMC4725485

[10] Zelko IN Mariani TJ Folz RJ . Superoxide dismutase multigene family: a comparison of the CuZn-SOD (SOD1), Mn-SOD (SOD2), and EC-SOD (SOD3) gene structures, evolution, and expression. Free Radic Biol Med. 2002;33(3):337 349. (10.1016/s0891-5849(02)00905-x) 12126755

[11] Teoh ML Fitzgerald MP Oberley LW Domann FE . Overexpression of extracellular superoxide dismutase attenuates heparanase expression and inhibits breast carcinoma cell growth and invasion. Cancer Res. 2009;69(15):6355 6363. (10.1158/0008-5472.CAN-09-1195) 19602586 PMC2728021

[12] Kliment CR Tobolewski JM Manni ML Tan RJ Enghild J Oury TD . Extracellular superoxide dismutase protects against matrix degradation of heparan sulfate in the lung. Antioxid Redox Signal. 2008;10(2):261 268. (10.1089/ars.2007.1906) 17961072 PMC2289772

[13] O’Leary BR Fath MA Bellizzi AM , et al. Loss of SOD3 (EcSOD) expression promotes an aggressive phenotype in human pancreatic ductal adenocarcinoma. Clin Cancer Res. 2015;21(7):1741 1751. (10.1158/1078-0432.CCR-14-1959) 25634994 PMC4383686

[14] Zhang Y Lu X Zhang Y , et al. The effect of extracellular superoxide dismutase (SOD3) gene in lung cancer. Front Oncol. 2022;12:722646. (10.3389/fonc.2022.722646) 35356201 PMC8959130

[15] Kim J Mizokami A Shin M , et al. SOD3 acts as a tumor suppressor in PC-3 prostate cancer cells via hydrogen peroxide accumulation. Anticancer Res. 2014;34(6):2821 2831. 24922645

[16] Griess B Tom E Domann F Teoh-Fitzgerald M . Extracellular superoxide dismutase and its role in cancer. Free Radic Biol Med. 2017;112:464 479. (10.1016/j.freeradbiomed.2017.08.013) 28842347 PMC5685559

[17] Liu X Xu Y Meng Q , et al. Proteomic analysis of minute amount of colonic biopsies by enteroscopy sampling. Biochem Biophys Res Commun. 2016;476(4):286 292. (10.1016/j.bbrc.2016.05.114) 27230957

[18] Yang R Wei L Fu QQ Wang H You H Yu HR . SOD3 ameliorates H2O2-Induced Oxidative Damage in SH-SY5Y Cells by Inhibiting the Mitochondrial Pathway. Neurochem Res. 2016;41(7):1818 1830. (10.1007/s11064-016-1897-x) 27084770

[19] Livak KJ Schmittgen TD . Analysis of relative gene expression data using real-time quantitative PCR and the 2(-Delta Delta C(T)) Method. Methods. 2001;25(4):402 408. (10.1006/meth.2001.1262) 11846609

[20] Chen J Wu Y Luo X , et al. Circular RNA circRHOBTB3 represses metastasis by regulating the HuR-mediated mRNA stability of PTBP1 in colorectal cancer. Theranostics. 2021;11(15):7507 7526. (10.7150/thno.59546) 34158864 PMC8210600

[21] Wang Z Zhou L Xiong Y , et al. Salinomycin exerts anti-colorectal cancer activity by targeting the β-catenin/T-cell factor complex. Br J Pharmacol. 2019;176(17):3390 3406. (10.1111/bph.14770) 31236922 PMC6692576

[22] Hu Y Zhou J Ye F , et al. BRD4 inhibitor inhibits colorectal cancer growth and metastasis. Int J Mol Sci. 2015;16(1):1928 1948. (10.3390/ijms16011928) 25603177 PMC4307342

[23] Huang Z Gan S Zhuang X , et al. Artesunate inhibits the cell growth in colorectal cancer by promoting ROS-dependent cell senescence and autophagy. Cells. 2022;11(16) (10.3390/cells11162472) PMC9406496 36010550

[24] Wang YN Zeng ZL Lu J , et al. CPT1A-mediated fatty acid oxidation promotes colorectal cancer cell metastasis by inhibiting anoikis. Oncogene. 2018;37(46):6025 6040. (10.1038/s41388-018-0384-z) 29995871

[25] Lin S Li Y Zamyatnin AA Jr Werner J Bazhin AV . Reactive oxygen species and colorectal cancer. J Cell Physiol. 2018;233(7):5119 5132. (10.1002/jcp.26356) 29215746

[26] Trachootham D Alexandre J Huang P . Targeting cancer cells by ROS-mediated mechanisms: a radical therapeutic approach? Nat Rev Drug Discov. 2009;8(7):579 591. (10.1038/nrd2803) 19478820

[27] Harris IS DeNicola GM . The complex interplay between antioxidants and ROS in cancer. Trends Cell Biol. 2020;30(6):440 451. (10.1016/j.tcb.2020.03.002) 32303435

[28] Marginean C Streata I Ioana M , et al. Assessment of oxidative stress genes SOD2 and SOD3 polymorphisms role in human colorectal cancer. Curr Health Sci J. 2016;42(4):356 358. (10.12865/CHSJ.42.04.04) 30581589 PMC6269622

[29] Mira E Carmona-Rodríguez L Pérez-Villamil B , et al. SOD3 improves the tumor response to chemotherapy by stabilizing endothelial HIF-2α. Nat Commun. 2018;9(1):575. (10.1038/s41467-018-03079-1) 29422508 PMC5805714

[30] Carmona-Rodríguez L Martínez-Rey D Fernández-Aceñero MJ , et al. SOD3 induces a HIF-2α-dependent program in endothelial cells that provides a selective signal for tumor infiltration by T cells. J Immunother Cancer. 2020;8(1) (10.1136/jitc-2019-000432) PMC7319787 32591431

[31] Singh B Bhat HK . Superoxide dismutase 3 is induced by antioxidants, inhibits oxidative DNA damage and is associated with inhibition of estrogen-induced breast cancer. Carcinogenesis. 2012;33(12):2601 2610. (10.1093/carcin/bgs300) 23027624 PMC3510741

[32] Teoh-Fitzgerald ML Fitzgerald MP Jensen TJ Futscher BW Domann FE . Genetic and epigenetic inactivation of extracellular superoxide dismutase promotes an invasive phenotype in human lung cancer by disrupting ECM homeostasis. Mol Cancer Res. 2012;10(1):40 51. (10.1158/1541-7786.MCR-11-0501) 22064654 PMC3262094

[33] Smith MJ Culhane AC Killeen S , et al. Mechanisms driving local breast cancer recurrence in a model of breast-conserving surgery. Ann Surg Oncol. 2008;15(10):2954 2964. (10.1245/s10434-008-0037-5) 18622646

[34] Györffy B Lanczky A Eklund AC , et al. An online survival analysis tool to rapidly assess the effect of 22,277 genes on breast cancer prognosis using microarray data of 1,809 patients. Breast Cancer Res Treat. 2010;123(3):725 731. (10.1007/s10549-009-0674-9) 20020197

[35] Ahmadiankia N Khosravi A . Significance of epithelial-to-mesenchymal transition inducing transcription factors in predicting distance metastasis and survival in patients with colorectal cancer: A systematic review and meta-analysis. J Res Med Sci. 2020;25:60. (10.4103/jrms.JRMS_174_19) 33088297 PMC7554549

[36] Cao H Xu E Liu H Wan L Lai M . Epithelial-mesenchymal transition in colorectal cancer metastasis: a system review. Pathol Res Pract. 2015;211(8):557 569. (10.1016/j.prp.2015.05.010) 26092594

[37] Sabouni E Nejad MM Mojtabavi S , et al. Unraveling the function of epithelial-mesenchymal transition (EMT) in colorectal cancer: metastasis, therapy response, and revisiting molecular pathways. Biomed Pharmacother. 2023;160:114395. (10.1016/j.biopha.2023.114395) 36804124

[38] Sun YL Bai T Zhou L , et al. SOD3 deficiency induces liver fibrosis by promoting hepatic stellate cell activation and epithelial-mesenchymal transition. J Cell Physiol. 2021;236(6):4313 4329. (10.1002/jcp.30174) 33230845

[39] Gorowiec MR Borthwick LA Parker SM Kirby JA Saretzki GC Fisher AJ . Free radical generation induces epithelial-to-mesenchymal transition in lung epithelium via a TGF-β1-dependent mechanism. Free Radic Biol Med. 2012;52(6):1024 1032. (10.1016/j.freeradbiomed.2011.12.020) 22240154

[40] Sibenaller ZA Welsh JL Du C , et al. Extracellular superoxide dismutase suppresses hypoxia-inducible factor-1α in pancreatic cancer. Free Radic Biol Med. 2014;69:357 366. (10.1016/j.freeradbiomed.2014.02.002) 24509158 PMC3981470

[41] Laatikainen LE Castellone MD Hebrant A , et al. Extracellular superoxide dismutase is a thyroid differentiation marker down-regulated in cancer. Endocr Relat Cancer. 2010;17(3):785 796. (10.1677/ERC-10-0021) 20576801

[42] Teoh-Fitzgerald ML Fitzgerald MP Zhong W Askeland RW Domann FE . Epigenetic reprogramming governs EcSOD expression during human mammary epithelial cell differentiation, tumorigenesis and metastasis. Oncogene. 2014;33(3):358 368. (10.1038/onc.2012.582) 23318435 PMC3711965

